# The Climatic Treatment of Phthisis

**Published:** 1897-10-02

**Authors:** 


### Medical Progress and Hospital Clinics.

[*The Editor will be glad to receive offers of co-operation and contributions from members of the profession. All letters should be addressed to* The Editor, at the Office, 28 & 29, Southampton Street, Strand, London, W.C.]

### THE CLIMATIC TREATMENT OF PHTHISIS.

In the various discussions which took place at Moscow on the treatment of tuberculosis, taking the term in its narrower sense as meaning phthisis, there seemed to be practical unanimity as to the immense importance of climate as a therapeutic agent. The question is, What climate?

Professor V. Leyden said that the assumed specific action of fresh air in tuberculosis was one of the myths of medicine. It had no such action, and its value was a purely hygienic one, strengthening the body and rendering it more resistant to the attacks of pathogenic organisms. By living in the open air and in airy apartments, and by sleeping with open windows, a hardening process was induced by which the diseased body acquired such resisting power as not only to tolerate the disease but to overpower it. The air, he said, should be pure, as free as possible from dust, and not liable to great and sudden changes of temperature.

The claims of high altitudes were advocated by Dr. von Ziemssen, Dr. Senator, and others. Dr. Ziemssen said that it was clear that patients who went to high altitudes got a great deal of fresh air. One could see patients sitting in the sun drinking their morning coffee with snow all around them, yet the sunbeams were so warm that they were perfectly comfortable. But the fresh air was not all. The altitude excited the blood-making organs and increased the corpuscular elements in the blood. There were, however, contra indications. A tuberculous patient with fever should never be sent to a height, for he would certainly be made worse and his fever increased. Almost all patients without fever did well, and even fever, if wholly due to pleurisy or bronchitis, was not a contra indication. Advanced cases, however, should be kept as near home as possible. Dr. Senator added pharyngeal and laryngeal cases to the list of those which should not be sent to high altitudes.

Dr. Vivant, of Monte Carlo, pointed out that the bacillus of tuberculosis resisted both high and low temperatures alike, so that no climate could claim absolute immunity. Those climates which were of most use in the treatment of phthisis had certain characters in common, viz., purity and relative dryness. Wind was injurious, because wind caused dust which was impurity. For the same reason, viz., the impurity of the air, large cities should be avoided. So far as concerned its influence on the respiratory tract, temperature of air was of little importance, yet there was an advantage on the side of heat, in that cold climates exposed feeble people to various circulatory troubles which were best avoided. The advantage of the Mediterranean resorts was that, along with pure air and plentiful solar radiation, they were less cold, permitting the patient to live in the open air continually in thin, light clothes. Our impression, however, is that people on the Riviera are as faddy about the times when they may go out as they are anywhere else, while the light clothes they feel compelled to wear when the air is warm are a serious source of danger.

